# Levelling up as a fair solution in AI enabled cancer screening

**DOI:** 10.3389/fdgth.2025.1540982

**Published:** 2025-02-25

**Authors:** Sahar Abdulrahman, Markus Trengove

**Affiliations:** GSK.ai, GSK, King’s Cross, London, United Kingdom

**Keywords:** AI, equity, fairness, screening, cancer, inequalities

## 1 Introduction

With the proliferation of artificial intelligence (AI) enabled tools in healthcare, clinicians have raised concerns about the potential for bias and subsequent negative impacts on underrepresented groups (1). The causes of bias in model deployment are multifaceted and can occur throughout the model development process (2). A well-recognized example within model training includes unrepresentative datasets that limit model generalizability in real-world populations (3). Whilst AI may outperform current standards of care for well-represented groups, models can perform worse for under-represented groups (4–9). Diversifying datasets is the obvious solution to bias caused by homogenous training data, however data collection is a long term project that may take years or even decades to acquire (10). The dilemma for policymakers currently is that releasing unfair tools can harm under-represented groups, whilst withholding them would cause significant welfare opportunity costs for well-represented groups. To address this problem, bioethicists like Vandersluis and Savalescu (11) have suggested alternate deployment strategies such as “selective deployment”, which would deploy AI tools only to well-represented groups. This incurs an obvious fairness cost. The issue of fairness in diagnostic testing is not specific to AI applications and debates also remain ongoing in the field of mainstream medicine on how to address this challenge (12, 13). By examining the case study of faecal immunochemical test (FIT) screening, which has been shown to perform more effectively for male patients in detecting bowel cancer, this paper supports the use of sex adjustment to “level up” female patients (14). Through this example within mainstream medicine, lessons learned from real-world policy can be transferred to clinical AI deployment which will be illustrated using a parallel case study of AI-assisted breast cancer screening.

## 2 Bowel cancer screening

**Figure 1:**
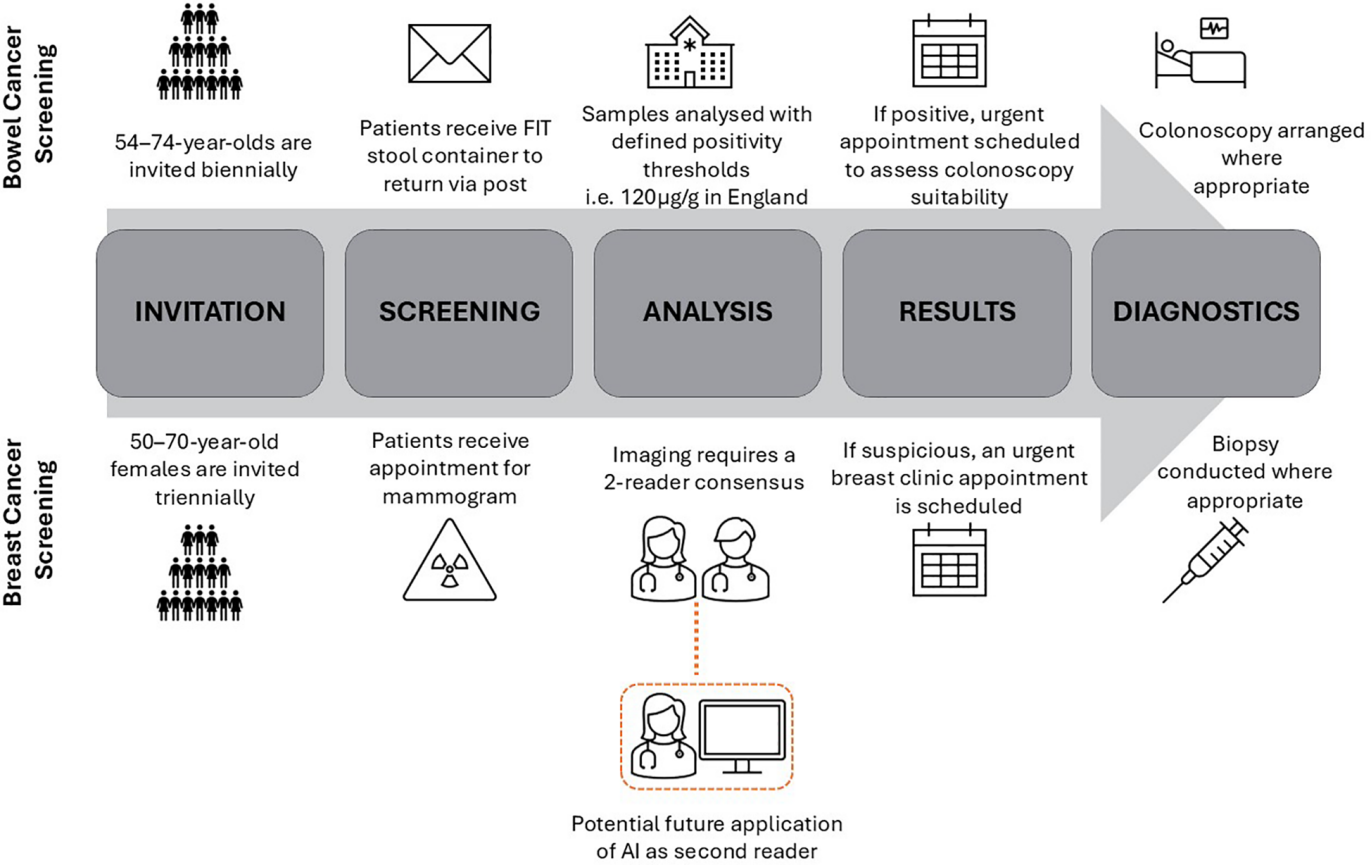
NHS clinical workflows of parallel case studies (bowel and breast national screening).

National Health Service (NHS) England introduced FIT screening for bowel cancer detection in 2019, see Figure 1 for summary of clinical workflow (15). FIT tests are stool samples that measure the concentration of blood in the stool, and if above a specified threshold triggers referral for further investigation which is usually a colonoscopy. In the UK, the National Screening Committee (NSC) set the FIT threshold (120 µg/g) based on cost-effectiveness analysis, where effectiveness is determined by Quality-Adjusted Life Years (QALY) gained, and system capacity (16). A lower FIT threshold results in more “positive” tests, with a subsequent greater rate of unnecessary colonoscopies. Conversely, higher FIT thresholds will result in a lower burden on colonoscopy constraints at the risk of more missed cancers. There is increasing evidence that FITs perform worse for female patients, who have a lower median faecal blood measurement than males (17). For each FIT threshold, cancer detection rates have been found to be lower in the female subgroup (18). Despite this, the UK continues to use universal FIT thresholds in contrast to some other countries who have adopted sex-adjusted thresholds resulting in a positive test at a lower blood concentration in female patients (19). For example, Sweden's thresholds for positivity of 40 µg/g and 80 µg/g for females and males respectively, has resulted in more equal proportions of cancer detection in subgroups but at the cost of higher rates of negative colonoscopies (20). Similar trends have also been seen in Finland who have sex-adjusted thresholds (21).

## 3 Discussion

A cost-benefit analysis of sex-adjusted FIT thresholds will be conducted. The aim of which is to assess the effectiveness of “levelling up” through sex adjustment in ensuring equitable health outcomes when “unfair” diagnostic tests are being utilised. Following this, using a parallel case study of breast cancer screening, the way in which levelling up can be applied to AI deployments, such as AI assisted mammogram interpretation, will be explored. These case studies will illustrate the usefulness of transfer learning from mainstream medicine to the emerging field of algorithmic fairness.

### 3.1 Cost-benefit analysis

In the case of bowel screening, a lower FIT threshold for female patients is the fairest strategy for maximal utility. From a public health perspective, increased detection of cancer for female patients at levels similar to men has the potential to reduce overall bowel cancer mortality and morbidity (22). Economically, by reducing the false negative rate in female patients, earlier detection of cancer can be more cost effective as earlier presentations will be more amenable to treatment which is particularly important in a publicly funded health system (23). In turn, overall health service costs are reduced by first line treatments, and reduced social care costs associated with advanced cancer. Health gains will vary between countries due to differences in underlying population risk, but evidence from Sweden shows that nearly 25% of female patients who would have been classified as negative by universal thresholds, were subsequently diagnosed with bowel cancer as a result of the lower sex-adjusted thresholds (19). From an ethical standpoint, lower thresholds for female patients acknowledges that universal processes may be suboptimal in ensuring fair outcomes, particularly in medicine where much of the evidence base is grounded on white male normativity (24).

The costs of levelling up disadvantaged subgroups centre on the impacts of more false positives. Firstly, the clinical risk posed by higher rates of false positives will differ depending on application, and whilst colonoscopies are not completely free of harm they do constitute a relatively lower risk intervention. For example, a randomised trial exploring the effect of colonoscopy screening found that of approximately 12,000 patients who had a colonoscopy, there were no bowel perforations or deaths within the 30 days post procedure (25). Furthermore, specialist nurses screen all patients with a positive FIT test before colonoscopy to ensure they are fit enough for the procedure as a further safety net to mitigate harm (26). Secondly, a significant cost in levelling up is the effect on system capacity as healthcare providers may not be able to provide necessary colonoscopies due to constraints of unit space, equipment and qualified personnel (27). Some may argue that in this case it would be equitable to increase the threshold for male patients whilst keeping the female threshold the same as this could lead to equivalent performance between subgroups without exceeding existing colonoscopy capacity. Although it may lead to more similar outcomes, it is widely accepted that down-levelling the male group by increasing the existing missed cancer rate would be unethical. Instead, healthcare policymakers must mandate subgroup analysis prior to deployment in a responsible by design approach, so that appropriate thresholds can be set with both subgroup performance and system constraints being considered.

### 3.2 Healthcare specific challenges

Levelling up in healthcare presents unique challenges specific to clinical medicine. Although this paper advocates for adjustments to mitigate for poor performance in subgroups, it acknowledges that there is a need for post-deployment evaluation due to the complex nature of disease manifestation. Data drift refers to changes in the properties of data over time from what was used in model training (28). Although FIT tests are not enabled by AI, a phenomena similar to data drift can occur whereby changes in disease can mean FIT threshold are no longer appropriate; for example, bowel cancer presenting more in younger patients and declining incidence in men (29). Therefore, thresholds should not be eternally fixed and regular post-deployment evaluation should be conducted to ensure that thresholds are continuing to be fit for purpose and meeting the ever-changing needs of patients.

Whilst FIT testing disadvantages female patients, there are other subgroups who are also not well-represented in both clinical trial and training data, such as non-white racial groups, who would benefit from levelling up in other contexts. Race-adjustment may be more difficult to adopt than sex-adjustment due to controversies surrounding race-based medicine stemming from historically exploitative practices such as Sims’ experimentation on enslaved black women (30). This paper acknowledges that race is social construct and is also critical of the historic underpinnings of how race categories were and continue to be defined (31). However, race-adjustment can be a useful tool in addressing health inequality in instances where it is used to uplevel groups who receive inadequate care due to system failures, in part due to the impacts of systemic racism, rather than to propagate the belief of innate biological differences. When levelling up poorly performing subgroups with adjustment, transparency will be critical in ensuring understanding and trust in healthcare providers, particularly in groups who have faced historic injustice.

### 3.3 Conditions for levelling up

Though adjustment can be a useful tool to mitigate for differential performance in subgroups, this should not be seen as one-size-fits-all solution. Rather, adjustment is intended to add to existing research on strategies for deploying such models, allowing for a comprehensive guide that policymakers can draw from. There are specific conditions under which adjustment is the most suitable approach. For this mitigation strategy to be effective there needs to be subgroup analysis that identifies a negative bias, specifically an underdiagnosis. Furthermore, adjustment will result in higher rates of false positive results for subgroups and deploying teams must understand the clinical sequalae of over referral. A false positive in different clinical contexts will have different repercussions, which can also be the case with the same application deployed across separate NHS trusts who have varying guidelines. Adjustment is preferred in contexts where there is a low-risk intervention, with high gain such as in the case of cancer screening. Next, workflows where there is a human-in-the-loop will mitigate harm of over-referral, such as the specialist nurse contact to assess fitness for colonoscopy in FIT testing.

An example of how these conditions can apply to clinical AI deployment includes the use of AI in the parallel case study of breast cancer screening. Similarly to FIT testing, mammograms are offered as a screening test in a national cancer screening programme in the NHS, see Figure 1 for clinical workflow. The use of AI in imaging, also known as computer vision, is the most popular application of AI in the health service (32). Despite the NSC finding a lack of evidence to introduce AI in NHS breast screening, countries like Sweden have already begun trialing AI as a second reader of mammograms in prospective studies (33, 34). Recent research has highlighted that a commercially available model diagnosing suspicious lesions from mammogram images overpredicts suspicious lesions in the images of black patients (35). Despite the concerns that this research has raised, there is a context in which this could be a harm mitigation strategy. An intentional higher false positive rate in black patients could be an example of levelling up if there was an initial underdiagnosis bias in this subgroup. Levelling up would be suitable given the high gain of possible cancer detection and low risk due to the double read requirement on mammograms in national screening (36). The existing workflow acts to reduce risk by ensuring one of the readers is a clinician-in-the-loop who can query AI diagnoses and seek a third reader opinion if necessary. Furthermore, if this safety net fails (i.e., both AI and human reader wrongly classify as suspicious), an urgent specialty review is organised to decide if a biopsy is necessary further lowering the risk to patients.

Levelling up doesn't solve the reasons why models may perform differentially, but does offer a solution in how to mitigate for harm through use of the clinical workflow. The causes of algorithmic bias are multifaceted and can happen at each point in the model development pipeline. As such, cross functional teams including developers, clinicians and researchers must attempt to elicit such causes and act together to highlight possible interventions to counteract preventable root causes. An example of such interventions includes initiatives to engage with underrepresented groups in data collection efforts and emerging techniques such as the use of synthetic data to diversify datasets (37–39).

## 4 Summary

In summary, levelling up can be an approach that safely balances fairness and utility when certain conditions are met. The parallel case studies highlights the usefulness of transfer learning from mainstream medicine, where solutions to unfair diagnostics have a real-world evidence base, to clinical AI. Whilst levelling up is a useful strategy to mitigate harm, it is essential that there remains a focus on addressing preventable root causes of algorithmic bias.
